# Oral fluids for detection and characterization of *Salmonella* in commercial finisher pigs

**DOI:** 10.3389/fvets.2026.1876360

**Published:** 2026-09-04

**Authors:** Mariah Post, Fernando L. Leite, Phillip Gauger, Nubia Macedo, Mariamawit Z. Mohammed, Thinh Tran Pham Tien, Isadora M. P. Coelho, Isadora F. Machado, Eduardo F. Costa, Gustavo S. Silva

**Affiliations:** 1 Department of Veterinary Diagnostic and Production Animal Medicine, College of Veterinary Medicine, Iowa State University, Ames, IA, United States; 2 Boehringer Ingelheim Animal Health USA Inc., Duluth, GA, United States; 3 Department of Epidemiology, Bioinformatics and Animal Models, Wageningen Bioveterinary Research, Lelystad, Netherlands

**Keywords:** fecal sample, oral fluids, *Salmonella*, surveillance, swine

## Abstract

*Salmonella* remains an important pathogen in swine production due to its impact on animal health and its role in foodborne illnesses. However, routine surveillance in pigs relies largely on fecal sampling, which can be labor-intensive and difficult to scale across large production systems. This study aimed to develop and evaluate a novel approach for monitoring *Salmonella* in finisher pigs using oral fluids in combination with sequential enrichment, culture, and polymerase chain reaction (PCR). A cross-sectional study was conducted at three commercial finisher sites in Iowa between February and May 2025, where paired oral fluid and pooled fecal samples were collected from 51 pens. The samples were processed using sequential enrichment followed by bacteriological culture and multiplex polymerase chain reaction to detect and characterize *Salmonella*. Direct PCR testing of raw samples did not detect *Salmonella* in either sample type. However, tests performed after enrichment successfully detected the pathogen in both samples, demonstrating the importance of enrichment for molecular detection in oral fluids. The culture of enriched oral fluids yielded viable isolates suitable for serotyping, and molecular testing enabled the rapid identification of the two serotypes most relevant to swine and public health (Typhimurium and I 4,[5],12:i:-). *Salmonella* was detected in 86.3% of the pens using at least one method, and oral fluids demonstrated diagnostic performance comparable to pooled fecal samples. These findings establish that oral fluids, when combined with sequential enrichment, are a practical and scalable sample type for monitoring *Salmonella* in finisher pigs.

## 1 Introduction

*Salmonella* is a common Gram-negative bacterium widely distributed in the environment and has remained a persistent challenge in swine populations for decades, contributing to both animal health concerns and food safety risks (1). The genus *Salmonella* comprises two species, *Salmonella enterica* and *Salmonella bongori*, with *S. enterica* being responsible for nearly all infections in pigs and humans (2). Within *S. enterica*, subspecies *enterica* (subspecies I) is predominant in warm-blooded hosts and accounts for the majority of serovars associated with swine colonization and human salmonellosis (3).

Pigs can harbor a diverse range of *Salmonella* serovars, often without demonstrating clinical signs, although infections may also result in enteric or systemic diseases depending on host and pathogen factors (4, 5). Several serovars, including Typhimurium and I 4,[5],12:i:-, are repeatedly identified in swine populations and are of particular public health relevance due to their role in pork contamination and human illness (1, 6, 7).

Monitoring of pig carcasses has traditionally been used to assess contamination at slaughterhouses; however, it has limited sensitivity for detecting the overall presence of *Salmonella* in swine production systems (8). In Europe, *Salmonella* prevalence in pig carcasses during official inspection is, on average, 3.15% but can reach up to 15% compared with 1.51% under food business operator monitoring (9). In Brazil, federal inspections in 2014–2015 found *Salmonella* spp. on 10.0% of pig carcasses before chilling and 4.6% after chilling (10). Although these results provide indicators of contamination at slaughterhouses, carcass monitoring alone does not capture the wider epidemiology of *Salmonella* in pigs and pork products (3, 11). Studies from South America also demonstrate widespread detection in live pigs; for example, *Salmonella* was isolated from 23.47% of 848 samples submitted for diagnosis in Colombia between 2022 and 2023 (12). Together, findings from Europe, North America, and South America reinforce the global importance of swine in *Salmonella* epidemiology and support the need for harmonized surveillance and control strategies across production systems.

This is particularly important because *Salmonella* infections in pigs can negatively affect herd productivity and pose a significant food safety concern. Even in the absence of overt clinical symptoms, infected grow–finish pigs may exhibit reduced growth performance and feed efficiency compared to non-infected animals (13). Subclinical infections are particularly relevant in commercial systems because colonized pigs may intermittently shed *Salmonella* while remaining clinically normal, contributing to pathogen persistence and transmission within and between herds (14). These impacts on productivity and pathogen circulation highlight the importance of effective monitoring and surveillance strategies in swine populations (1, 5, 8, 15).

Traditional bacteriological culture remains the primary method for detecting *Salmonella* in swine, as it provides viable isolates required for serotyping and further characterization. Polymerase chain reaction (PCR)-based methods offer faster detection but still require further evaluation for routine use in swine surveillance and are often used as screening tools that may require enrichment or culture confirmation (7). Since *Salmonella* is shed primarily through feces, fecal samples are considered an appropriate matrix for confirming the presence of the pathogen at the animal level and have been widely used in diagnostic and epidemiological investigations in swine populations (16). In practice, fecal samples are commonly collected as fecal pooled (FP) samples from the pen floor to represent the infection status of a group of pigs. Pooled sampling can improve detection sensitivity compared to single-animal samples by increasing the probability of capturing feces from infected pigs and accounting for intermittent shedding patterns (17). Oral fluids (OFs) consist of aggregated saliva and oral secretions collected from pigs using suspended cotton ropes that they chew due to natural exploratory behavior (18). This non-invasive, welfare-friendly, pen-based method has been widely validated for pathogen surveillance under field conditions, including porcine reproductive and respiratory syndrome virus (PRRSV) (19, 20), swine influenza virus (SIV) (21), and *Mycoplasma hyopneumoniae* (MHYO) (22–24), More recently, OFs have also been evaluated for the detection of *Lawsonia intracellularis* (25). However, previous reports on the use of OFs for *Salmonella* have primarily focused on antibody detection, while evidence supporting their use for direct detection and isolation of the organism remains limited (26, 27).

Therefore, the objectives of this study were as follows: first, to develop and evaluate approaches for detecting *Salmonella* in oral fluid (OF) samples from finisher pigs using both culture and real-time PCR. Second, to assess the feasibility of recovering and characterizing *Salmonella* serotypes from OF using adapted culture methods and compare the serotypes identified in OF with those detected in FP samples. Finally, culture results of both sample types were used to estimate the probability of *Salmonella* detection under field conditions and to evaluate the agreement between sample types, while a Bayesian latent class analysis was applied to compare OF and FP samples as sampling methods because no perfect gold standard diagnostic test was available, allowing the estimation of test performance while accounting for uncertainty in the true infection status.

## 2 Methods

### 2.1 Study design

A cross-sectional observational study was conducted between February and May 2025 to evaluate the probability of *Salmonella* detection in OF and FP samples under commercial conditions for monitoring finishing pig populations. Samples were collected at three commercial sites in Iowa, each housing approximately 1,000 pigs across 48 pens (≈22 pigs/pen). Pigs were approximately 11 weeks old at the time of sampling. Before enrollment, each site was prescreened for *Salmonella* shedding using pooled fecal samples collected from six pens. For each prescreened pen, five fresh fecal samples were collected from different locations across the pen floor where fecal material had accumulated and combined into a single pooled sample of approximately 25 g. Each pooled sample was enriched and tested by PCR, and the sites were considered eligible if at least one of the six pen-level pooled fecal samples tested positive. Only *Salmonella*-positive herds meeting the defined inclusion criteria were enrolled in the study.

### 2.2 Sample size calculation

The sample size was determined using McNemar’s test to ensure sufficient power to detect a difference in *Salmonella* detection between paired OF and FP samples collected from the same pen (28). Assuming 95% confidence, 80% power, and an expected difference in detection probability of 70% versus 90% between the paired sampling methods, a minimum of 51 paired OF–FP samples was required. These detection probabilities were selected *a priori* to represent a conservative 20% difference between sampling methods rather than being derived from previously reported detection rates. This sample size provides adequate power to assess differences in *Salmonella* detection between paired OF and FP samples collected from the same pen under field conditions. In our study, this corresponded to 17 pens per site across 3 sites (51 pens and 102 total samples), with the sample size divided equally among sites because they had similar numbers of pens and animals. Each selected pen was sampled once.

### 2.3 Animal care and use

The samples used in this study were collected as part of routine herd health surveillance within a commercial swine production system. The sample collection was performed by a responsible company veterinarian following the sampling protocol provided for the study in accordance with the farm’s standard management and animal care practices. No experimental procedures or additional animal handling beyond routine management were required; therefore, the study was considered exempt from additional animal ethics review.

### 2.4 Herd selection and inclusion criteria

Sites were selected based on prior *Salmonella* infection history and production similarity to ensure comparable environmental, housing, and management conditions. Eligible sites were commercial finisher sites (10–11 weeks of age at the time of sampling) with documented clinical *Salmonella* cases in their previous production lots. Additional inclusion criteria required that facilities be of similar size and design, house approximately 1,000 pigs across 48 pens, and have not administered any live *Lawsonia intracellularis* or *Salmonella* vaccine to the current group of pigs to prevent interference with *Salmonella* detection (4, 29, 30). Before enrollment, a pre-screening step was conducted to confirm active *Salmonella* shedding within each site. For this purpose, six pens per site were selected, and paired OF and FP samples were collected. The FP samples consisted of 25 g of fresh feces collected from five different floor locations within each pen (adapted from 16), while the OF samples were obtained following the rope-chewing protocol. All samples underwent double enrichment using Rappaport–Vassiliadis (RV) and Tetrathionate (TTB) broths, followed by PCR testing. The sites were considered eligible for the main study if at least one sample tested positive for *Salmonella* by PCR (7).

### 2.5 Sample collection

#### 2.5.1 Oral fluid sampling

The OFs were collected in accordance with standard rope-chewing protocols (22). In each selected pen, a 3-strand twisted unbleached cotton rope (12 mm diameter; Skydog Rigging Equipment, Lake in the Hills, IL, USA) was suspended at shoulder height for 30–45 min. After pig interaction, the ropes were placed in single-use plastic bags, and approximately 10 mL of the fluid was manually transferred into sterile 50 mL conical tubes (Thermo Fisher Scientific, Pittsburgh, PA, USA).

#### 2.5.2 Pooled fecal sampling

From each corresponding pen, five fresh fecal samples were collected from different floor locations across the pen floor where fecal material had accumulated using 5 mL sterile Whirl-Pak® spoons and combined (~25 g total) into sterile Whirl-Pak® bags (Filtration Group, Pleasant Prairie, WI, USA). Five locations were sampled to obtain the recommended 25 g pooled sample while improving the spatial representation of the pen and increasing the likelihood of including fecal materials from multiple pigs. This approach was adapted from the pooled fecal sampling methodology described by Arnold and Cook (16). Both FP and oral OF samples were stored in a cooler and transported to the Iowa State University Veterinary Diagnostic Laboratory (ISU-VDL, Ames, IA) on the same day as collection.

### 2.6 Sample processing and aliquoting

At the Iowa State University Veterinary Diagnostic Laboratory (ISU-VDL, Ames, IA), each original sample was homogenized and aliquoted as follows:

- Aliquots for PCR were prepared from raw samples consisting solely of fecal material or OF: 0.1 g of feces was transferred to 1 mL of phosphate-buffered saline (PBS), vortexed to homogenize, and 1 mL of OF was pipetted directly into sterile tubes. These “raw” aliquots were submitted for DNA extraction before enrichment.
- Aliquots for culture: 25 g of FP or 10 mL of OF were processed following the *Salmonella* culture Method 2 of Love and Rostagno (31). This method was selected because it demonstrated the highest sensitivity for *Salmonella* recovery from swine fecal samples among the culture protocols evaluated.

#### 2.6.1 Culture and enrichment

Culture was performed using tetrathionate broth (TTB) (BD Difco, Franklin Lakes, NJ, USA) and Rappaport-Vassiliadis (RV) (BD Difco, Franklin Lakes, NJ, USA) sequential enrichment, as described by method two in Love and Rostagno (31). Briefly, the samples were enriched at a 1:10 proportion. For FP, 25 g of feces were added to 250 mL of TTB, which was then incubated at 37 °C for 48 h. For OF, 10 mL of the sample was added to 100 mL of TTB and incubated under the same conditions. For OF samples with an available volume below the expected amount due to leakage during transport (*n* = 7; available volumes ranging from 1.0 to 7.0 mL), the volume of tetrathionate broth (TTB) was adjusted to achieve a standardized final enrichment volume of 100 mL. Following the first enrichment, 10 mL of the TTB culture was transferred to 100 mL of Rappaport–Vassiliadis (RV) broth and incubated at 37 °C for 24 h.

After RV broth incubation and prior to plating, the broth was briefly mixed to homogenize, and 1 mL of aliquot was transferred to a sterile microcentrifuge tube and centrifuged at approximately 1,500 × *g* (4,000 rpm) for 5 min. The supernatant was discarded, and the resulting bacterial pellet (“culture pellet”) was retained and stored at −80 °C for subsequent molecular analyses.

Following incubation, 10 μL of RV broth was streaked onto xylose-lysine-tergitol-4 (XLT-4) (BD Difco, Franklin Lakes, NJ, USA) and incubated aerobically at 37 °C for 24 h. Plates were read for *Salmonella*-suspect colonies, which appeared as round colonies with a black center and typically a yellowish periphery, although red or pink peripheries could also be observed. Two suspect colonies per sample were selected for isolation and identification. When only one suspected colony was present, that colony was selected. Colonies were confirmed using the MALDI-TOF MS (Bruker Daltonics, Billerica, MA). Confirmed *Salmonella* spp. isolates were held on slants and submitted to the National Veterinary Services Laboratories (NVSL, Ames, IA) for serotyping.

#### 2.6.2 Molecular and serotyping testing

All “raw” and “culture-pellet” aliquots were tested for *Salmonella* DNA by multiplex real-time PCR (7). The multiplex PCR assay differentiates *Salmonella* spp., *S. typhimurium*, and I 4,[5],12:i:-;. This assay targets the invA gene (universal *Salmonella* marker) and the fliA, fljB, and hin-iroB regions to differentiate serovars *S. typhimurium* and S. 4,[5],12:i:-. PCR reactions (25 μL) were prepared using the QuantiTect Virus + ROX Kit (Qiagen, Waltham, MA) and run under the following cycling conditions: 20 min at 50 °C, 5 min at 95 °C, followed by 40 cycles of 15 s at 95 °C and 60 s at 60 °C. DNA extraction was performed according to the manufacturer’s instructions for the assay kit, and Ct interpretation thresholds were applied as previously described for this multiplex real-time PCR assay (7). Samples were considered positive when the cycle threshold (Ct) value was < 34 and interpretable at the serovar level when it was < 30. Each batch included positive and negative extraction and amplification controls.

### 2.7 Statistical analysis

All statistical analyses were performed in R (32). A significance level of 0.05 was considered statistically significant, where applicable.

#### 2.7.1 Probability of detection analysis

To address the study objective of assessing the probability of *Salmonella* detection using OF relative to FP under field conditions, logistic mixed-effects regression models were used, using the package lme4 (33). The detection outcome (positive/negative) was modeled as the binary response variable, with sample type (OF vs. FP) included as the primary explanatory variable. The site was included as a random intercept to account for clustering of samples within production systems. The model was specified as Result ~ SampleType + (1 | Farm). Model results were used to estimate the probability of *Salmonella* detection for each sample type, with corresponding 95% confidence intervals.

#### 2.7.2 Agreement between OF and FP detection results

The agreement between OF and FP was first evaluated to describe the concordance in *Salmonella* detection outcomes. Paired OF and PF results were summarized into 2 × 2 contingency tables separately for culture and PCR outcomes. Cohen’s kappa statistic was calculated to quantify agreement beyond chance (34). In addition, Holley and Guilford’s G and Gwet’s AC1 were calculated because these coefficients are recognized as robust agreement estimators for 2 × 2 contingency tables, particularly in the presence of imbalanced data structures (35). All 95% confidence intervals for agreement statistics were estimated using 10,000 bootstrap resamples.

#### 2.7.3 Bayesian modeling

A Bayesian latent class model (BLCM) was implemented to estimate the diagnostic performance of OF and FP cultures for the detection of *Salmonella* in the absence of a gold-standard sample type. Latent class analysis is a statistical approach used to estimate the sensitivity and specificity of diagnostic tests and disease prevalence when a perfect reference test is unavailable (36, 37). The Bayesian framework allows for the incorporation of prior knowledge about disease prevalence or test performance and accounts for uncertainty in parameter estimation (38).

The model was specified for two diagnostic tests across three populations represented by the three farms included in the study, allowing farm-level prevalence to vary across populations. Paired OF and FP culture results were summarized into 2 × 2 contingency tables for each farm and modeled using multinomial likelihood.

The global posterior prevalence, calculated as the simple average of the farm-specific posterior prevalence estimates, was approximately 0.71. Test sensitivities and specificities were assumed to be constant across farms. Informative beta-prior distributions were assigned to test accuracy parameters based on biological plausibility and the level of prior knowledge available for each sample type. For pooled fecal culture, sensitivity was assigned a Beta(7,5) prior, reflecting the expectation that FP culture would have moderate-to-high sensitivity, given its established role in *Salmonella* detection. For OF culture, sensitivity was assigned a Beta(5,7) prior, reflecting the expectation of moderate sensitivity and the more limited prior information available for culture recovery from OF. Specificity priors for both FP and OF cultures were specified as Beta(25,3), reflecting biologically plausible high specificity for culture-based detection with confirmatory identification of isolates. Conditional independence between the two tests was assumed, given the latent infection status.

The model was implemented in R using the rjags package (39). Three Markov Chain Monte Carlo (MCMC) chains were run with an adaptation phase of 5,000 iterations followed by a burn-in period of 10,000 iterations. Posterior samples were obtained from 300,000 iterations with a thinning interval of 100. Posterior summaries were reported as posterior medians and 95% credible intervals (CrI) for test sensitivity, specificity, and farm-level prevalence. Model convergence was assessed by visual inspection of trace plots, posterior density plots, and Gelman–Rubin diagnostics.

Posterior predictive values were derived from the posterior distributions of sensitivity, specificity, and prevalence. For each posterior sample, the posterior prevalence estimates from the three farms were averaged to obtain an overall prevalence estimate. Positive predictive value (PPV) and negative predictive value (NPV) were then calculated separately for each sample type as:

PPV=Se×Prev/[(Se×Prev)+((1−Sp)×(1−Prev))]

NPV=Sp×(1−Prev)/[((1−Se)×Prev)+(Sp×(1−Prev))]

where Se represents sensitivity, Sp represents specificity, and Prev represents the simple average posterior prevalence across farms (34). Posterior summaries for PPV and NPV were reported as medians with 95% credible intervals.

## 3 Results

### 3.1 Descriptive results

A total of 51 pens across three commercial finisher sites were sampled, yielding 102 paired OF and FP samples. *Salmonella* was detected in 42 of 51 pens (82.3%) by at least one of the culture and enrichment-PCR methods (Table 1). Detection frequencies varied by site, with higher detection at Sites 2 (33.3%) and 1 (31.4%) and lower detection at Site 3 (17.6%). When data were combined across sites, culture detected *Salmonella* in 34 of 51 OF samples (66.7%) and 38 of 51 FP samples (74.5%). PCR testing was used to evaluate *Salmonella* detection in both sample types before and after the enrichment process used for culture. Direct PCR performed on unenriched OF and FP aliquots did not detect *Salmonella* DNA in any samples (Ct > 34). However, PCR performed after enrichment (TTB–RV) detected *Salmonella* DNA in 57 of 100 samples (57.0%), as two enriched samples from Site 1 were lost before testing. Following enrichment, the PCR identified *Salmonella* in 36 of 51 FP samples (70.6%) and 32 of 49 OF samples (65.3%). These findings indicate that PCR detection of *Salmonella* in both OF and FP samples was only achieved after enrichment, highlighting the importance of the enrichment step for improving molecular detection of these sample types.

**Table 1:** Detection of *Salmonella* by PCR (raw and enriched) and culture by site and sample type.

Site	‘Raw’ qPCR		Enriched qPCR		Culture	
	FP	OF	FP	OF	FP	OF
1	0/17	0/17	15/15*	16/17	14/17	14/17
2	0/17	0/17	17/17	14/17	17/17	14/17
3	0/17	0/17	9/17	4/17	7/17	4/17

FP, pooled fecal samples; OF, oral fluids. *Two samples lost in the process.

### 3.2 Serotyping and PCR results

A total of 59 *Salmonella*-positive cultures (from both OF and FP samples) were submitted to the National Veterinary Services Laboratories (NVSL) for serotyping. Distinct serotype profiles were identified at the three sites (Table 2).

**Table 2:** Distribution of *Salmonella* serotypes identified from culture-positive pooled fecal (FP) and oral fluid (OF) samples across three study sites, with total positive (n) indicating total serotype recoveries and n (%) indicating the frequency of each serotype within each sample type and site.

Site	Sample type	Total positive (n)	Serotype	*n* (%)
Site 1	FP	14	Rissen	7 (50%)
Site 1	FP	14	I 4,[5],12:i:-	5 (36%)
Site 1	FP	14	Agona	2 (14%)
Site 1	OF	14	Rissen	6 (43%)
Site 1	OF	14	I 4,[5],12:i:-	5 (36%)
Site 1	OF	14	Agona	3 (21%)
Site 2	FP	17	I 4,[5],12:i:-	17 (100%)
Site 2	OF	14	I 4,[5],12:i:-	14 (100%)
Site 3	FP	7	Senftenberg	4 (57%)
Site 3	FP	7	Johannesburg	3 (43%)
Site 3	OF	4	Senftenberg	3 (75%)
Site 3	OF	4	Johannesburg	1 (25%)

Enriched PCR identified *Salmonella* in 36 of 51 FP samples (70.6%) and 32 of 49 OF samples (65.3%). As stated previously in the Methods section, the multiplex PCR assay (7) differentiates *Salmonella* spp., *S. typhimurium*, and I 4,[5],12:i:-; therefore, other serotypes such as Rissen, Agona, Senftenberg, and Johannesburg are only identified at the genus level (*Salmonella* spp.) without further serovar discrimination. These results confirmed that enrichment substantially increased the analytical sensitivity of PCR, particularly for OF samples.

### 3.3 Probability of detection and agreement

**Figure 1:**
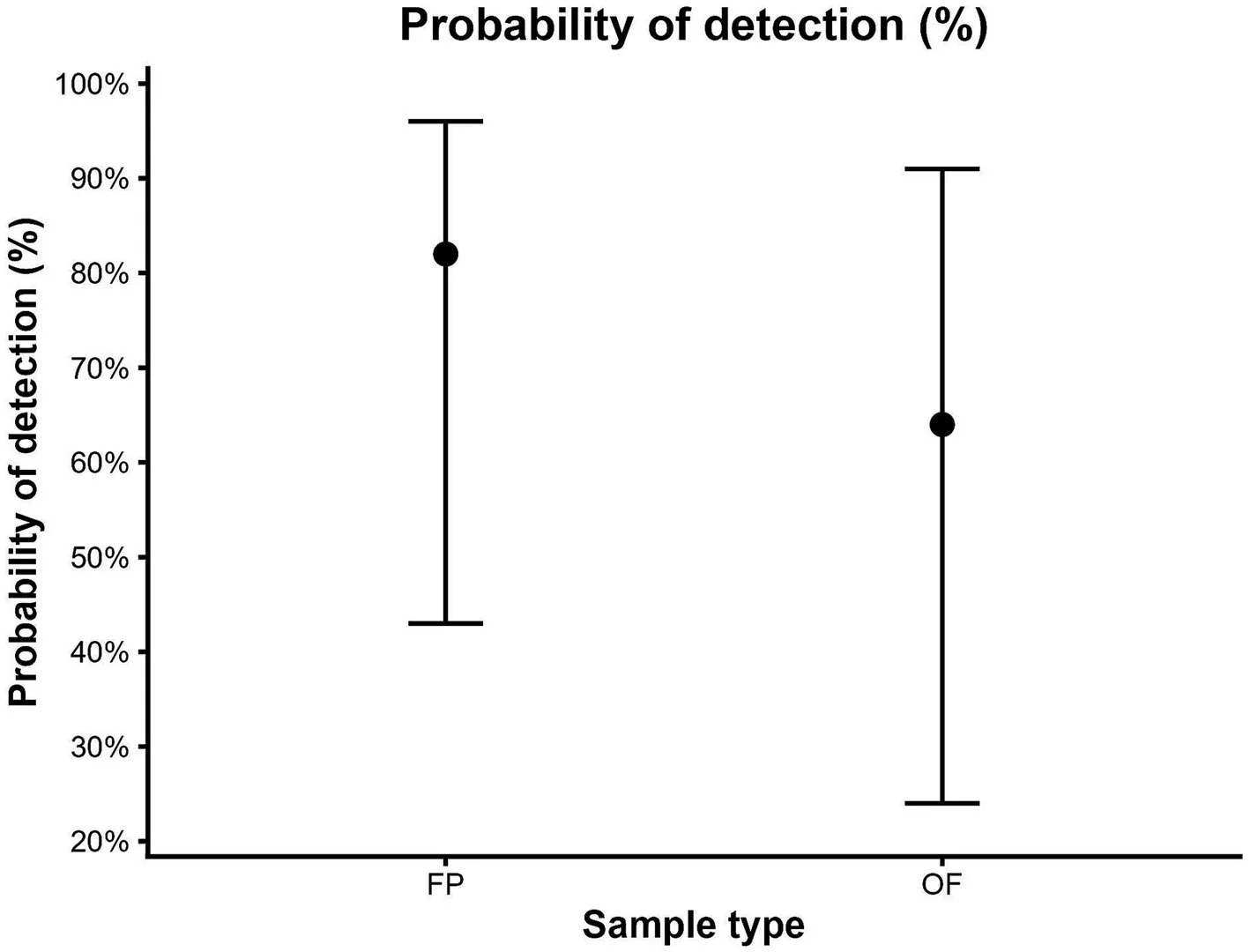
Probability of detection (%) of *Salmonella* by sample type. FP, pooled fecal samples; OF, oral fluids. Error bars represent 95% confidence intervals.

A logistic regression analysis indicated no statistically significant difference in the probability of detection between OF and FP (*p* = 0.13). The estimated probability of detection was 81.8% (95% CI: 43.3–96.4%) for FP and 63.8% (95% CI: 23.7–90.9%) for OF (Figure 1).

When comparing paired oral fluid (OF) and pooled fecal (FP) samples at the pen level, agreement between sample types was positive. Based on Cohen’s kappa, OF culture and FP culture showed fair agreement (κ = 0.373; 95% CI: 0.094–0.617), with an overall concordance of 72.5% (95% CI: 58.8–84.3%). The OF culture versus FP culture comparison included all 51 pens, whereas PCR-based comparisons included 49 paired observations because two samples were unavailable for PCR testing. When all farms were combined, the agreement between OF and FP cultures was moderate, based on Holley and Guilford’s G and Gwet’s AC1, with estimates of 0.451 (95% CI: 0.176–0.686) and 0.518 (95% CI: 0.264–0.739), respectively.

Agreement between OF PCR and FP PCR was slightly higher than agreement between OF culture and FP culture, with a Cohen’s kappa of 0.434 (95% CI: 0.167–0.689), an overall concordance of 77.6% (95% CI: 65.3–87.8%), a Holley and Guilford’s G of 0.551 (95% CI: 0.306–0.755), and a Gwet’s AC1 of 0.638 (95% CI: 0.398–0.832). Agreement between culture and enriched PCR results within the same sample type was higher than agreement between OF and FP sample types. Among FP samples, the agreement between culture and PCR was substantial based on Cohen’s kappa (κ = 0.805; 95% CI: 0.557–1.000), with 93.9% overall concordance (95% CI: 85.7–100.0%). For OF samples, agreement between culture and PCR was almost perfect (κ = 0.867; 95% CI: 0.697–1.000), with 93.9% overall concordance (95% CI: 85.7–100.0%). Similarly, Holley and Guilford’s G and Gwet’s AC1 indicated high agreement between culture and PCR within each sample type. For OF samples, the culture versus PCR agreement showed a G of 0.878 (95% CI: 0.714–1.000) and an AC1 of 0.887 (95% CI: 0.749–1.000). For FP samples, culture versus PCR agreement showed a G of 0.878 (95% CI: 0.714–1.000) and an AC1 of 0.911 (95% CI: 0.793–1.000) (see Table 3).

**Table 3:** Agreement between oral fluids (OF) and pooled pen fecal (FP) samples and between culture and enriched PCR results for *Salmonella* detection.

Comparison	No. of paired pens	Cohen’s κ (95% CI)	Overall concordance, % (95% CI)	Holley and Guilford’s G (95% CI)	Gwet’s AC1 (95% CI)
OF culture vs. FP culture	51	0.373 (0.094–0.617)	72.5 (58.8–84.3)	0.451 (0.176–0.686)	0.518 (0.264–0.739)
OF PCR vs. FP PCR	49	0.434 (0.167–0.689)	77.6 (65.3–87.8)	0.551 (0.306–0.755)	0.638 (0.398–0.832)
OF culture vs. OF PCR	49	0.867 (0.697–1.000)	93.9 (85.7–100.0)	0.878 (0.714–1.000)	0.887 (0.749–1.000)
FP culture vs. FP PCR	49	0.805 (0.557–1.000)	93.9 (85.7–100.0)	0.878 (0.714–1.000)	0.911 (0.793–1.000)

95% CI, 95% confidence intervals; OF, oral fluids; FP, pooled fecal samples; PCR, polymerase chain reaction.

### 3.4 Bayesian latent class model

The Bayesian latent class model was applied to estimate the diagnostic performance of the sample types, as no gold-standard sample type exists for *Salmonella* detection. Posterior estimates indicated that pooled fecal samples had higher estimated sensitivity than oral fluids, whereas oral fluids had higher estimated specificity. The estimated sensitivity was 0.84 (95% CrI: 0.72–0.93) for pooled fecal samples and 0.71 (95% CrI: 0.57–0.83) for oral fluids, while the estimated specificity was 0.84 (95% CrI: 0.70–0.95) and 0.91 (95% CrI: 0.79–0.98), respectively. The global posterior prevalence was estimated at 0.70 (95% CrI: 0.64–0.78). At this prevalence, PPV was high for both pooled fecal samples (0.93; 95% CrI: 0.85–0.98) and oral fluids (0.95; 95% CrI: 0.88–0.99), whereas NPV was lower for pooled fecal samples (0.69; 95% CrI: 0.52–0.84) and oral fluids (0.57; 95% CrI: 0.41–0.72) (see Table 4).

**Table 4:** Posterior estimates from the Bayesian latent class model for *Salmonella* detection using OF and FP.

Parameter	PF	OF
Global posterior prevalence, median (95% CrI)	0.70 (0.64–0.78)	0.70 (0.64–0.78)
Sensitivity, median (95% CrI)	0.84 (0.72–0.93)	0.71 (0.57–0.83)
Specificity, median (95% CrI)	0.84 (0.70–0.95)	0.91 (0.79–0.98)
PPV, median (95% CrI)	0.93 (0.85–0.98)	0.95 (0.88–0.99)
NPV, median (95% CrI)	0.69 (0.52–0.84)	0.57 (0.41–0.72)

CrI, credible interval; OF, oral fluids; PF, pooled fecal samples; PPV, positive predictive value; NPV, negative predictive value.

## 4 Discussion

This study evaluated the performance of OF and FP for the detection and characterization of *Salmonella* in finisher swine under commercial conditions. The comparable detection and serotype characterization obtained from both sample types support the epidemiological value of OF as a practical tool for pen-level *Salmonella* surveillance. A central methodological contribution of this study was the successful adaptation of an enrichment-based culture protocol (31), traditionally applied to fecal samples, to OF, along with PCR following enrichment. In the present study, *Salmonella* DNA was not detected when PCR was applied directly to raw OF or FP. In contrast, PCR performed after sequential enrichment consistently detected *Salmonella* in both sample types, demonstrating that enrichment is critical for increasing analytical sensitivity and enabling molecular detection. These findings highlight that the detection of *Salmonella* in both sample types is strongly dependent on the use of appropriate enrichment procedures (7), and that combining enrichment with PCR provides a viable approach for surveillance using this sample type.

Across all sites, both sample types yielded substantial pen-level *Salmonella* positivity. Although FP showed slightly higher culture detection than OF, the difference was not statistically significant, and both sample types identified similar serotype distributions. Agreement was moderate between OF and FP and high between culture and enriched PCR within each sample type. Bayesian latent class modeling indicated higher estimated sensitivity for FP culture and higher estimated specificity for OF culture, although credible intervals overlapped substantially, suggesting similar performance for pen-level surveillance under field conditions. These findings support previous studies demonstrating that OF integrates pig-origin and environmental material and can detect enteric pathogens despite intermittent or heterogeneous shedding within a pen (18, 20). However, unlike several viral pathogens for which direct PCR of OF provides adequate detection (38), *Salmonella* was not detected by PCR in raw OF or FP from these subclinical populations. Detection was achieved after enrichment, likely because selective enrichment suppresses competing microbiota, allowing *Salmonella* to multiply and increase target DNA above the assay’s limit of detection, thereby reducing high Ct values that may compromise PCR sensitivity and interpretation (7, 31). Similar sensitivity challenges have been reported for *Lawsonia intracellularis* when pathogen exposure or within-pen prevalence is low (40), indicating that enrichment may be particularly important for bacterial surveillance in subclinical populations.

Oral fluids have occasionally been criticized for lower culture yields due to reduced bacterial viability in this sample type (41), but the present study demonstrates that when culture is successful, OFs provide serotype profiles comparable to feces. This finding is particularly relevant in light of studies showing that traditional culture methods often fail to detect minority serovars, thereby masking the true complexity of *Salmonella* communities within pens (42).

The PCR results on enriched samples provide additional insights into the diagnostic behavior of these sample types. When PCR was performed on samples lacking enrichment, detection was absent, whereas samples enriched twice for *Salmonella* were detected. These findings mirror observations from earlier studies showing that PCR post-enrichment can help reconcile discrepancies between culture and molecular detection, particularly for surveillance purposes or when evaluating environmental or population sample types (7, 41, 43). The fact that enrichment increased detection in both sample types reinforces the importance of incorporating enrichment methods for bacterial targets, when available, before performing PCR, as demonstrated in this study for *Salmonella* surveillance.

Fecal samples primarily reflect active shedding by pigs, whereas oral fluids also capture indirect exposure through environmental contamination on ropes (20). This distinction likely explains why the agreement was not perfect and why each sample type detected some pens that the other did not. Within-sample type agreement between culture and enriched PCR was high, suggesting that the majority of discrepancies were due to low-level contamination rather than false positives. These patterns parallel findings from both swine and cattle surveillance studies, which show that pen-level sampling tools (ropes, composite feces, environmental swabs) often produce partially overlapping, but distinct, detection patterns (16, 17, 44).

The Bayesian latent class analysis provided a refined assessment of sample type performance by accounting for the absence of a gold standard. We chose to compare oral fluids to pooled pen fecal samples because pooled pen fecal samples have been described as being more sensitive than individual pig samples and they also allow for an increase in sample volume, which is also associated with the sensitivity of *Salmonella* detection, as fecal sample weight is directly correlated with the probability of detection (16, 17, 45). Differences in farm-level prevalence influence the predictive values of each sampling method. Because the latent class model was based on a limited number of farms and informative prior distributions, posterior estimates should be interpreted as exploratory and conditional on the model assumptions. Pooled feces showed slightly higher sensitivity, but the posterior distributions overlapped substantially, indicating that these differences were not meaningful at the population level. However, oral fluids exhibited higher specificity. This may reflect differences in how each sample type captures population-level exposure. Pooled fecal sampling provided a representative composite sample of the pen rather than target pigs with a known shedding status. Because the individual shedding status was unknown, the 25-g pooled sample could include fecal material from both shedding and non-shedding pigs. In contrast, oral fluids were collected through active rope chewing and may therefore reflect more direct, behavior-driven exposure to circulating *Salmonella*, although they can also include contributions from non-shedding pigs. These results are consistent with the broader literature on pen-based sampling, in which composite samples often show high sensitivity at the expense of specificity due to environmental carryover, while oral fluids exhibit more selective capture of pathogens through pig–rope interactions (18, 46). Therefore, our findings support the interpretation that oral fluids provide a conservative yet reliable estimate of pen-level *Salmonella* status.

The similarity of serotypes identified in oral fluids and pooled feces further supports the utility of oral fluids for population-level characterization of *Salmonella*. Across all sites, both sample types consistently identified the same dominant serovars, including I 4,[5],12:i:-, Rissen, Senftenberg, Agona, and Johannesburg. These serovars are frequently reported in finisher pigs and have been documented in multiple North American and European studies (41, 47).

Taken together, these results have important implications for herd-level *Salmonella* surveillance. Oral fluids offer numerous operational advantages, including reduced labor, minimal animal disturbance, and the ability to scale sampling across large populations without requiring individual handling (19, 48). When paired with enrichment PCR, oral fluids provide high sensitivity and reliable serotype representation, making them well-suited to both routine monitoring and targeted epidemiologic investigations. In addition to improving detection, our results demonstrate the feasibility of recovering *Salmonella* isolates from oral fluids using adapted culture methods, enabling serotype characterization directly from this sample type. This is particularly relevant given the study objective of comparing serotypes identified in oral fluids and pooled fecal samples and supports the use of oral fluids not only for detection but also for epidemiologically meaningful characterization. Also, PCR-based serotyping enables rapid identification of key *Salmonella* serotypes without the delays associated with traditional serotyping, which can take several weeks. This rapid turnaround is particularly valuable in field settings, where timely information on the presence of important serotypes for swine health and food safety can support faster decision-making and response. Although pooled feces may retain advantages in very low-prevalence settings or when isolated recovery for downstream characterization is required, including serotyping, antimicrobial susceptibility testing (AST), and whole-genome sequencing (WGS), our findings indicate that oral fluids can complement conventional fecal sampling and, depending on the surveillance objective and validated testing protocol, may represent a practical alternative. Integrating oral fluids into surveillance frameworks may therefore improve efficiency, reduce costs, and support more frequent or systematic monitoring.

This study has limitations that should be acknowledged. First, the high prevalence across sites limited our ability to evaluate diagnostic performance at very low prevalence levels, where pooled feces may outperform oral fluids. Additionally, the wide confidence intervals around the estimated detection probabilities could indicate limited precision; however, the overlapping intervals and non-significant comparison provided no evidence of a difference in detection probability between OF and FP. Second, rope interaction behaviors were not measured; however, differences in pig participation could influence oral-fluid yield and detection probability. Although the pens evaluated in this study had pig densities typical of many finisher oral-fluid studies (approximately 30 pigs per pen), commercial finisher operations often house substantially larger groups, frequently exceeding 100 pigs per pen. Higher pig densities could influence the rope interaction patterns, saliva contribution, and ultimately the representativeness of oral-fluid samples, and this should be considered when extrapolating these findings to other production settings (20). Additionally, this study focused exclusively on finisher pigs; therefore, the performance of oral-fluid sampling for *Salmonella* detection in younger populations, such as nursery pigs, was not evaluated and may differ due to behavioral and management factors. Finally, environmental conditions, pen design, and production system factors may modify the relative performance of each sample type, and these variables warrant further exploration in longitudinal or multi-system studies.

Overall, this study describes novel methods for detecting *Salmonella* in oral fluids. The primary contributions of this study include the successful adaptation of *Salmonella* enrichment culture to oral fluids, recovery of viable *Salmonella* isolates from this sample type, evaluation of diagnostic performance using Bayesian latent class analysis, and strong agreement in serotype distribution between oral fluids and pooled feces. When the described enrichment method is used, with the option of further detection by PCR, oral fluids can be a reliable, practical, and biologically meaningful sample for detecting *Salmonella* in finisher pigs. Importantly, in addition to demonstrating comparable molecular detection, we successfully cultured *Salmonella* from oral fluids and recovered serotypes that matched those found in pooled feces, confirming that oral fluids can support both bacteriologic isolation and serovar characterization, an ability not demonstrated previously in the peer-reviewed literature. This capacity to generate culture-positive isolates from oral fluids substantially enhances their value in surveillance programs, as it enables confirmatory testing, antimicrobial susceptibility evaluation, and detailed serotype profiling from a single, pen-based sample. Together with their high specificity and strong serotype agreement with feces, these results indicate that oral fluids can play a central role in pen-level *Salmonella* monitoring, particularly when paired with enrichment-PCR workflows. Integrating oral fluids into modern surveillance systems may therefore enhance the efficiency, scalability, and actionable value of *Salmonella* detection across large swine production systems.

## Data Availability

The raw data supporting the conclusions of this article will be made available by the authors, without undue reservation.
