# Supplementary material for: Oral fluids for detection and characterization of Salmonella in commercial finisher pigs
Source: Front Vet Sci. 2026 Sep 4;13:1876360. doi: 10.3389/fvets.2026.1876360 (PMC13585974; doi:10.3389/fvets.2026.1876360)
Supplement: Supplementary file 1 [file Supplementary_File_1.docx]

1. Illustration of ‘raw’ PCR testing.

2. Illustration of the culture methodology.

3. Illustration of bacterial pellet PCR.
